# Perinatal transmission of human papilomavirus DNA

**DOI:** 10.1186/1743-422X-6-83

**Published:** 2009-06-21

**Authors:** Renato L Rombaldi, Eduardo P Serafini, Jovana Mandelli, Edineia Zimmermann, Kamille P Losquiavo

**Affiliations:** 1 Diagnosis – Molecular Laboratory, University of Caxias do Sul, Caxias do Sul, Rio Grande do Sul, Brazil; 2 Pathology Medical Laboratory, Department of Health and Biomedical Science, University of Caxias do Sul, Caxias do Sul, Rio Grande do Sul, Brazil; 3 Biotechnology Institute, University of Caxias do Sul, Caxias do Sul, Rio Grande do Sul, Brazil; 4 Outpatient Clinic of Genital Pathology, Department of Clinical Medicine, University of Caxias do Sul, Caxias do Sul, Rio Grande do Sul, Brazil

## Abstract

The purpose was to study the perinatal transmission of human papillomavirus DNA (HPV-DNA) in 63 mother-newborn pairs, besides looking at the epidemiological factors involved in the viral DNA transmission. The following sampling methods were used: (1) in the pregnant woman, when was recruited, in cervix and clinical lesions of the vagina, vulva and perineal region; (2) in the newborn, (a) buccal, axillary and inguinal regions; (b) nasopharyngeal aspirate, and (c) cord blood; (3) in the children, buccal was repeated in the 4^th ^week and 6^th ^and 12^th ^month of life. HPV-DNA was identified using two methodologies: multiplex PCR (PGMY09 and MY11 primers) and nested-PCR (genotypes 6/11, 16, 18, 31, 33, 42, 52 and 58). Perinatal transmission was considered when concordance was found in type-specific HPV between mother/newborn or mother/child. HPV-DNA genital was detected in 49 pregnant women submitted to delivery. Eleven newborns (22.4%, n = 11/49) were HPV-DNA positive. In 8 cases (16.3%, n = 8/49) there was type specific HPV concordance between mother/newborn samples. At the end of the first month of life three children (6.1%, n = 3/49) became HPV-DNA positive, while two remained positive from birth. In 3 cases (100%, n = 3/3) there was type specific HPV concordance between mother/newborn samples. In the 6th month, a child (2%, n = 1/49) had become HPV-DNA positive between the 1^st ^and 6^th ^month of life, and there was type specific HPV concordance of mother/newborn samples. All the HPV-DNA positive children (22.4%, n = 11/49) at birth and at the end first month of life (6.1%, n = 3/49) became HPV-DNA negative at the age of 6 months. The HPV-DNA positive child (2%, n = 1/49) from 1^st ^to the 6^th ^month of life became HPV-DNA negative between the 6^th ^and 12^th ^month of life and one child had anogenital warts. In the twelfth month all (100%, n = 49/49) the children studied were HPV-DNA negative. A positive and significant correlation was observed between perinatal transmission of HPV-DNA and the immunodepression of maternal variables (HIV, p = 0.007). Finally, the study suggests that perinatal transmission of HPV-DNA occurred in 24.5% (n = 12/49) of the cases studied.

## Introduction

Sexual transmission of human papillomaviruses (HPV) is widely recognized as a cause of anogenital warts and cervical cancer[1,2]. Today, the plurality of HPV is well-established; more than 120 HPV types have been identified. Papillomaviruses are also suspected of playing a role in a subset of oropharyngeal cancers, in squamous cell skin cancers, and possibly also in esophageal cancers, though the evidence is as yet less convincing than that for cervical cancer[3].

HPV is a sexually transmitted disease common in adolescents and adults[4]. It is becoming increasingly clear that HPV-DNA can be acquired by non-sexual routes, and that one of these would be mother-child transmission during the perinatal period [5-8]. This vertical transmission of HPV-DNA presumably may occur when the fetus passes through the infected birth canal [9-12] or by ascending infection, especially after premature rupture of the amniotic membranes [5-7]. Several authors referred to the presence of HPV in the amniotic liquid[13], in fetal membranes[12], in nasopharyngeal aspirates of concepts born by cesarean section[9,11], in cord blood[14] suggesting that HPV-DNA contamination occurred before birth (intrauterine) by transplacental route[14]. The implications of these observations have not yet been clearly established. The non-concordance of type specific HPV between mother and newborn appears to suggest the existence of other transmission routes such as by phomites (contaminated instruments), contact between the child and people (interfamily, friends). In children, the vertical transmission of HPV-DNA was related to juvenile recurrent respiratory papillomatosis[15] and to genital warts[16].

Therefore a prospective study was proposed in pregnant women with molecular diagnosis of genital HPV-DNA, as well as their newborns, in order to seek a better understanding of the risks of perinatal transmission of DNA virus, and also to study a few maternal variables that might be correlated with DNA virus transmission.

## Materials and methods

### Population studied

Between April 2005 and December 2008, a cross-sectional, prospective study was performed on pregnant women with a prior history of HPV infection, or who had abnormal Papanicolaou smear or genital warts, due to the high probability that they could have HPV infection. The women were referred from the Obstetrical Service of the University of Caxias do Sul and by the Basic Health Units of the Single Health System of Caxias do Sul. This study was performed with the approval of the Ethics Committee in Research at the University of Caxias do Sul, and of the Editorial and Scientific Board of the General Hospital of Caxias do Sul, and did not present a conflict of interest. The Letter of Free and Informed Consent and the epidemiological evaluation tool were obtained from all the women by individual interviews during the obstetrical examinations. At the end of the study 63 pairs of mothers/newborns were included in the research.

### Epidemiological evaluation

The epidemiological study was performed taking the following variables into account: age, race, level of education, smoking, marital status, marital stability, history of immunodepression (HIV – acquired immunodeficiency syndrome), type of HPV lesion (genital warts, LGSIL – low-grade squamous intraepithelial lesions, HGSIL – high-grade squamous intraepithelial lesions), site of HPV lesion (cervical, vaginal, vulvar and perineal), numbers of types of HPV-DNA in maternal genital (single, double and multiple), gestational age at the time HPV infection was diagnosed (weeks), time of labor (minutes), time of amniotic membrane rupture (minutes), type of delivery (cesarean section, vaginal and vaginal with forceps) and HPV lesion at delivery (genital warts, LGSIL – low-grade squamous intraepithelial lesions, HGSIL – high-grade squamous intraepithelial lesions).

### Sampling methods

#### Maternal

The maternal genital samples were obtained during pregnancy, at the first visit, when the pregnant woman was recruited. The sample was obtained using a special brush for cytopathological sampling of the cervix, which was used for genital brushing in the following order: cervix and possible clinical and subclinical lesions of the vagina, vulva and perineal region. The brush was placed in a TE solution (Tris HCl, pH 7.5 – 10 mM; EDTA, 1 mM), and the material collected was kept frozen at -20°C, until the desoxyribonucleic acid (DNA) was extracted.

#### Newborns

In newborns, in the first minutes of life, buccal, body, nasopharyngeal aspirates and arterial blood were obtained from the umbilical cord samples.

#### Buccal and body

The swabs were collected in the first minutes of life, using the special brush for cytopathological sampling of the cervix, with which brushing was performed in the following order: buccal cavities, axillary and inguinal regions of the newborn. The brush was placed in a TE solution (Tris HCl, pH 7.5 – 10 mM; EDTA, 1 mM) and kept frozen at -20°C, until DNA was extracted.

Collection with a brush from the buccal of the newborn was repeated in the 4^th ^week and 6^th ^and 12^th ^month of life.

#### Nasopharyngeal aspirates

The distal extremity of the tracheal aspiration catheter (n°6 or 8, Sondas Descartáveis Mercosul^® ^Linha Sondas Descartáveis Mercosul^®^, Empresa CPL Medical's Produtos Médicos LTDA), used to aspirate the upper airways (nasopharyngeal) of the newborn immediately after birth, was removed. The distal extremity of the catheter (about 4 cm long) was cut and placed in TE solution (Tris HCl, pH 7.5 – 10 mM; EDTA, 1 mM), keeping it frozen at -20°C, until DNA was extracted.

#### Arterial cord blood

The sample was collected directly from one of the arteries of the cord using a 3 ml disposable syringe (27/5 needle) to obtain about 1 ml of fetal blood. The collection was performed after clamping the cord and complete delivery of the placenta and fetal membranes. The fetal blood was placed in a KMA type tube with EDTA and frozen at -20°C, until DNA was extracted.

### DNA extraction

DNA was extracted in the blood and tissue samples using the *Wizard Genomic DNA Purification Kit *(Promega), according to the manufacturer's specifications. In the brush samples, DNA was extracted using 600 μl of NaOH 50 mM, stirred in a vortex for 5–10 seconds and later incubated at 95°C for 5 minutes. The solution was then neutralized with 60 μl of Tris HCl pH 8.0 and kept in a freezer at -20°C, until it was submitted to the next stages.

After the DNA extraction methodology, the products were submitted to two different PCR methods for HPV-DNA identification and typing: PCR multiplex and type specific nested multiplex PCR.

### Amplification of the β-globin and HPV

The DNA samples obtained from the extraction methodology were amplified in multiplex PCR, and this was composed by the PCO4 oligonucleotides (CAA CTT CAT CCA CGT TCA CC) and GH20 (GAA GAG CCA AGG ACA GGT AC), which amplified the segment of 268 base pairs (pb) of the human β-globin gene, ensuring the qualification and quantification of DNA for HPV analysis, and by the PGM09 and PGMY11 oligonucleotides, which amplify a segment of 450 pb of a preserved region of gene L1 of *Human Papillomavirus*[17]. The thermocycler, model PTC100 (MJResearch, Watertown, Mass) was used for amplification; the parameters for denaturation, annealing and lengthening of the ribbons were the following: 95°C for 5 minutes, followed by 40 51°C cycles for 30 seconds, 55°C for 1 minute, 72°C for 1 minute and, finally, 72°C for 5 minutes. Negative and positive controls were included with all amplifications, and the negative control was constituted by all elements except genomic DNA; and the positive control was constituted by HPV-DNA type 16 previously typing (Diagnosis Molecular Laboratory of University of Caxias do Sul). Four μg of the molecular DNA of the DNA *φ X 174RF *HaeIII molecular weight marker were used. The presence or absence of HPV-DNA fragments and β-globin amplified from the oligonucleotides was analyzed in 1.5% agarose gel, in buffer TBE 0.5× with 0.3% ethidium bromide (0.1 mg/μL solution), under ultraviolet light.

### Viral typing

The HPV positive samples were submitted to a new type of PCR-specific for viral type identification. For this purpose the RFLP (Restriction Fragment Length Polymorphism) technique was used, according to the methodology described by Bernard et al. (1994)[18]. The amplified product was digested by the BamHl, Ddel, Haelll, HinfI, PstI, RsaI and SauAIII enzymes and analyzed by vertical electrophoresis in 4% polyacrylamide gel (20.3% acrylamide, 0.7 bisacrylamide, 0.07% ammonium persulphate, TBE 1× TEMED 0.7 μL/mL – GibcoBRL). The pGEM (PROMEGA) was used as a molecular weight marker. Later the samples in polyacrylamide gel were stained with silver nitrate and the fragments obtained compared to the prototypes described by Bernard et al. (1994)[18].

### Amplification by nested-PCR in region E6/E7 of the HPV

The nested multiplex PCR (NMPCR) assay combines degenerate E6/E7 consensus primers and type-specific primers for the detection and typing of HPV genotypes 6/11, 16, 18, 31, 33, 35, 39, 42, 43, 44, 45, 51, 52, 56, 58, 59, 66 and 68. As to sensitivity and performance with clinical samples, the novel NMPCR assay is a potentially useful tool for HPV-DNA detection in epidemiologic and clinical follow-up studies, especially when accurate HPV typing and the detection of multiple HPV infections are required.

The samples were amplified in the first PCR reaction using the degenerated primers GP-E6-3F (GGG WGK KAC TGA AAT CGG T), GP-E6-5B (CTG AGC TGT CAR NTA ATT GCT CA) and GP-E6-6B (TCC TCT GAG TYG YCT AAT TGC TC), W being A/T; K, G/T; R, A/G; Y, C/T and N, A/C/G/T. These primers amplify a region of 630 pb of the E6/E7 region of the 38 most common types of HPV. The nested-PCR reaction is specific and was performed for types 6/11, 16, 18, 31, 33, 42, 52 and 58, which represent the viral types that are most prevalent in the region[19]. The primers used and the sizes of the amplified products are listed in table 1. The entire procedures, both the first reaction (PCR) and the second reaction (nested-PCR) occurred according to Sotlar et al., 2004[20].

**Table 1:** Sequences of type-specific nested PCR primers used in this study.

**HPVgenotype**	**Primer sequences**	Amplicon (pb)*
6/11	TGC AAG AAT GCA CTG ACC AC TGC ATG TTG TCC AGC AGT GT	334
16	CAC AGT TAT GCA CAG AGC TGC CAT ATA TTC ATG CAA TGT AGG TGT A	457
18	CAC TTC ACT GCA AGA CAT AGA GTT GTG AAA TCG TCG TTT TTC A	332
31	GAA ATT GCA TGA ACT AAG CTC G CAC ATA TAC CTT TGT TTG TCA A	263
33	ACT ATA CAC AAC ATT GAA CTA GTT TTT ACA CGT CAC AGT GCA	398
42	CCC AAA GTA GTG GTC CCA GTT A GAT CTT TCG TAG TGT CGC AGT G	277
52	TAA GGC TGC AGT GTG TGC AG CTA ATA GTT ATT TCA CTT AAT GGT	229
58	GTA AAG TGT GCT TAC GAT TGC GTT GTT ACA GGT TAC ACT TGT	274

* Base pairs.

### Perinatal transmission of HPV-DNA

In the study, perinatal transmission of HPV-DNA was considered when HPV type-specific agreement was observed between the samples mother/newborn or mother/child.

### Statistical analysis

Statistical analyses were performed with the SPSS computer software package (version 12.0 for Windows). Frequency tables were analyzed by using the chi-square test, with Pearson and likelihood ratio tests for the significance of differences between the categorical variables. The 95% confidence interval (95% CI) was calculated where appropriate. Differences in the means of continuous variables between the groups were analyzed by using nonparametric tests. In all analyses, probability values of < 0.05 were regarded as significant.

## Results

The genital HPV-DNA was detected in 49 women of the 63 who underwent delivery (mean age 23.9 ± 8 years; 14–41 years). The distribution of the viral types identified in the maternal genital samples are shown in table 2. Among the HPV-DNA positive (HPV-DNA+) cases, 54.9%, 1.2%, 40.2% and 3.7% were considered high risk, possible high risk, low risk and unclassified DNA, respectively[21]. The HPV-DNA detected most frequently were types 6/11 (20.7%), 42 (15.9%), 16 (15.9%), 18 (11%), 58 (6.1%) and 31, 35 and 52 (3.7% each). The numbers of types of HPV-DNA identified in maternal genital were single, double and multiple in 38.8%, 30.6% and 30.6% of the cases, respectively.

**Table 2:** HPV types in maternal genital sample.

HPV-DNA			
Type n = 18	Carcinogenic risk	Frequency n = 82	%

6/11	LR	17	20.7
42	LR	13	15.9
16	HR	13	15.9
18	HR	9	11
58	HR	5	6.1
31	HR	3	3.7
35	HR	3	3.7
52	HR	3	3.7
51	HR	2	2.4
54	LR	2	2.4
59	HR	2	2.4
26	PHR	1	1.2
33	HR	1	1.2
34	HR	1	1.2
45	HR	1	1.2
68	HR	1	1.2
70	LR	1	1.2
73	HR	1	1.2
NC*	-	3	3.7

The HPV types were identified by both multiplex PCR and nested multiplex PCR methods.*NC = HPV-DNA positive but could not be classified by type. LR – Low-risk HPV genotypes (HPV type 6, 11, 40, 42, 43, 44, 54, 61, 70, 72, 81 and CP6108); HR – High-risk HPV genotypes (HPV type 16, 18, 31, 33, 35, 39, 45, 51, 52, 56, 58, 59, 68, 73 and 82); PHR – Probable high-risk HPV genotypes (HPV type 26, 53 and 66)[21].

When analyzing the samples obtained from buccal and body scrapings, nasopharyngeal aspirate and arterial cord blood obtained during the first minutes of life, it was observed that 11 newborns (NB) (22.4%, n = 11/49) were positive for the research of HPV-DNA (Table 3). Of the HPV-DNA positive cases, 54.9%, 1.2%, 40.2% and 3.7% were types considered a high carcinogenic risk, possible high carcinogenic risk, low carcinogenic risk and non-classified DNA, respectively. Of this total of NB HPV-DNA+, 6 (12.2%, n = 6/49) were HPV-DNA+ in samples obtained by scraping the buccal and body, 5 (10.2%, n = 5/49) from the nasopharyngeal aspirate and 3 (6.1%, n = 3/49) in arterial cord blood. Concordance of type specific HPV was seen between the mother/NB in 16.3% (n = 8/49) of the cases. There was type specific HPV concordance between the maternal genital sample and the samples of the buccal and body scraping, nasopharyngeal aspirate and arterial cord blood of the NB, of 83.3% (n = 5/6), 60% (n = 3/5) and 100% (n = 3/3), respectively. Of the HPV-DNA+ cases, 66.7% and 33.3% were types considered a low and high carcinogenic risk, respectively. The types of HPV-DNA detected were: 6/11 (53.3%); 42, 18, 52 (13.3% each); and 59 (6.7%). One NB (n = 1/11, 9.1%) had HPV-DNA+ for two different types of virus.

**Table 3:** Clinical and laboratory history of genital HPV infection during pregnancy and delivery and distribution of HPV types in maternal, newborns and children in 1^st ^and 6^th ^and 12^th ^month of life samples.

	**Maternal epidemiology**				**HPV type in samples**						
	**Pregnancy**		**Delivery**		**Maternal**	**Newborn**			**Children**		

									**Buccal**		
									
**Case**	**HPV lesion type**	**HPV lesion site**	**Type**	**HPV lesion**	**Genital**	**Nasopharyngeal aspirates**	**Buccal and body**	**Cord blood**	**1^st ^month of life**	**6^th ^month of life**	**12^th ^month of life**

1	Warts	VV	V	No	6/11						
2	HGSIL	C	C	No	16+6/11		6/11		6/11		
3	HGSIL	C	C	Yes	16+31						
4	Warts	VV	V	No	16+42+54		42				
5	LGSIL	C	C	Yes	18						
6	Warts	VV	C	No	6/11+16+31						
7	Warts	C+VV+VG	V	No	6/11+42	6/11	6/11				
8	HGSIL	C	C	Yes	52	6/11					
9	LGSIL	C	V	Yes	42+51+NC*						
10	Warts	VV+VG	V	No	6/11						
11	LGSIL	C	V	No	18	18		18			
12	Warts	VV	V+F	No	NC*						
13	LGSIL	C	C	Yes	6/11+42						
14	LGSIL	C	C	Yes	16+42+58	59					
15	Warts	C+VV+VG	V	Yes	6/11+42						
16	Warts	VV+VG	C	No	6/11					6/11	
17	HGSIL	C	C	Yes	18+51						
18	Warts	VV+P	C	No	42+59						
19	Warts	VV	V	No	6/11						
20	HGSIL	C	C	No	42+35						
21	Warts	VV	C	Yes	52			52			
22	Warts	VV	C	Yes	34						
23	Warts	VV+VG	V	Yes	18						
24	Warts	VV	V	No	16+73						
25	Warts	VV+VG	C	No	68						
26	Warts	C	V	Yes	6/11+16				16		
27	Warts	C+VV+VG	V	No	16+58						
28	Warts	VV	C	Yes	6/11+33						
29	LGSIL	C	V	No	16						
30	LGSIL	C	V	Yes	52+42+58+54						
31	LGSIL	C	C	No	16						
32	Warts	VV	C	No	6/11						
33	HGSIL	C	V	Yes	18						
34	Warts	VV	V	Yes	11	11					
35	Warts	VV+VG	C	Yes	42		42				
36	LGSIL	C	V	Yes	16						
37	HGSIL	C	C	Yes	58		6/11+52		6/11		
38	HGSIL	C	V	No	6/11+18				6/11		
39	HGSIL	C	C	Yes	18+31						
40	Warts	VV	V	No	6/11						
41	Warts	VV	C	No	42+35+NC*						
42	LGSIL	C	C	Yes	42						
43	LGSIL	C	C	Yes	16+18+42						
44	LGSIL	C+VV+VG	C	Yes	18+26						
45	Warts	VV	V	Yes	6/11+58+59		6/11	6/11			
46	Warts	VV+VG	C	Yes	6						
47	Warts	VV	V	Yes	35						
48	Warts	VV	V	No	70						
49	Warts	VV+VG	V	No	6+45				6/11+52		
50	LGSIL	C	V	Yes	-						
51	Warts	VV	C	No	-						
52	HGSIL**	C	C	Yes	-						
53	Warts	VV+VG	C	Yes	-						
54	HGSIL	C	V	Yes	-						
55	LGSIL	C	V	Yes	-						
56	HGSIL	C	V	Yes	-						
57	HGSIL	C	V	No	-						
58	HGSIL	C	C	No	-						
59	HGSIL	C	C	Yes	-						
60	LGSIL	C	V	No	-						
61	HGSIL	C	V	Yes	-						
62	LGSIL	C	C	Yes	-						
63	HGSIL	C	V	Yes	-						

The HPV types were identified by both multiplex PCR and nested multiplex PCR methods.*NC = HPV-DNA positive but could not be classified by type. **Child had anogenital warts (HPV type 6/11) in the 12^th ^month of life.

Delivery type = C – cesarean section; V- vaginal; and V+ F – vaginal with forceps.

HPV lesion site = C – cervical; VG – vaginal; VV – vulva; P- perineal.

HPV lesion type = Warts – genital warts; LGSIL – low-grade squamous intraepithelial lesions; HGSIL – high-grade squamous intraepithelial lesions.

Studying the buccal samples of infant, obtained at the end of the first month of life, it was observed that five children (10.2%, n = 5/49) had HPV-DNA+ (Table 3). Three of these children (6.1%, n = 3/49) became HPV-DNA+ during the first month of life, while two were positive since birth. Concordance of the type specific HPV was observed between mother/NB in 100% (n = 3/3) of the new cases. Of the HPV-DNA+ cases, 66.7% and 33.3% were types considered low and high carcinogenic risk, respectively. The types of HPV-DNA detected were: 6/11 (53.3%), 16 and 52 (13.3% each). One NB (20%, n = 1/5) had HPV-DNA+ for two different types of virus. Nine (81.8%, n = 9/11) of the eleven children that were HPV-DNA+ at birth became HPV-DNA negative at the end of the first month of life.

Studying the buccal samples from infant, obtained in the 6^th ^month of life, it was observed that one child (2%, n = 1/49) had become HPV-DNA+ between the 1st and 6th month of life (Table 3). There was agreement of the type specific HPV between the mother/NB (HPV type 6/11). All the children who were HPV-DNA+ (22.4%, n = 11/49) at birth and at the end first month of life (6.1%, n = 3/49) became HPV-DNA negative at the age of 6 months.

Studying the buccal samples of infant, obtained in the 12^th ^month of life, all the children (30.6%, n = 15/49) who were HPV-DNA+ from birth to the 6^th ^month of life became HPV-DNA negative between the 6^th ^and 12^th ^month of life (Table 3).

One (1.6%, n = 1/63) child had anogenital warts in the 12^th ^month of life. The type of HPV-DNA detected was 6/11. In this case, in epidemiological maternal history, it was observed that the pregnant joined the research for high-grade squamous intraepithelial lesions, with negative result for HPV-DNA. This child was born by cesarian section without early rupture of membranes and by restricted fetal growth. The mother showed normal results in colposcopic and Papanicolaou smear evaluates in the 2nd, 5^th^, 8^th ^and 12^th ^months postpartum. The buccal samples of the child remained HPV-DNA negative during the 12 months of life.

Analyzing the demographic and behavioral factors (Table 4) a positive and significant correlation was found between the presence of HPV-DNA in the NB or child and maternal history of immunodepression (HIV, p = 0.007).

**Table 4:** HPV status of the newborn and children and maternal factors.

**Maternal variable**	**Newborn and children HPV-DNA**			
	
	**Positive** **(n = 12)**		**Negative** **(n = 37)**	
**Age (years)**				
≤ 19	6	(50%)	17	(45.9%)
≥ 20 to ≤ 29	3	(25%)	12	(32.4%)
≥ 30 to ≤ 39	3	(25%)	6	(16.2%)
≥ 40 to ≤ 49	-	-	2	(5.4%)
Mean for newborn HPV-DNA positive group (25.3 ± 8.1 years)	-	-	-	-
Mean for newborn HPV-DNA negative group (23.6 ± 8.2 years)	-	-	-	-
				
**Race**				
White	10	(83.3%)	34	(91.9%)
Non-white	2	(16.7%)	3	(8.1%)
				
**Level of education**				
Illiterate	-	-	-	-
Elementary (complete or incomplete)	7	(58.3%)	21	(56.8%)
High school (complete or incomplete)	4	(33.3%)	16	(43.2%)
College (complete or incomplete)	1	(8.4%)	-	-
				
**Smoking**				
No	7	(58.3%)	27	(73%)
< 10 cigarettes per day	1	(8.3%)	5	(13.5%)
≥ 10 cigarettes per day	4	(33.3%)	5	(13.5%)
				
**Marital status**				
Married	1	(8.3%)	10	(27%)
Single	3	(25%)	8	(21.6%)
Cohabiting	7	(58.4%)	18	(48.6%)
Divorced, separated	1	(8.3%)	1	(2.7%)
				
**Marital stability (years)**				
≤ 2	9	(75%)	25	(67.6%)
≥ 3 to ≤ 5	2	(16.7%)	8	(21.6%)
≥ 6	1	(8.3%)	4	(10.8%)
Mean for newborn HPV-DNA positive group (2.9 ± 4.6 years)	-	-	-	-
Mean for newborn HPV-DNA negative group (2.9 ± 4.7 years)	-	-	-	-
				
History of Immunodepression (HIV)*				
No	10	(83.3%)	37	(100%)
Yes	2	(16.7%)	-	-

Data are reported as number and percentage (in parentheses) of newborn and children infection positive or negative for human papillomavirus. *P < 0.007 indicates a statistically significant difference between the positive and negative groups by Pearson's chi-square test (HIV – acquired immunodeficiency syndrome).

The statistical analysis did not show any significant association between the presence of HPV-DNA in the NB or child and the other variables studied (Tables 4 and 5).

**Table 5:** HPV status of the newborn and children and delivery factors.

**Maternal variable**	**Newborn and children HPV-DNA**			
	
	**Positive** **(n = 12)**		**Negative** **(n = 37)**	
**Type of HPV lesion**				
Genital warts	7	(58.3%)	21	(56.8%)
LGSIL^1^	2	(16.7%)	10	(27%)
HGSIL^2^	3	(25%)	6	(16.2%)
				
**Site of HPV lesion**				
Uterine cervix	5	(41.7%)	16	(43.2%)
Vulva	4	(33.3%)	12	(32.4%)
Vulva + vagina	2	(16.7%)	5	(13.5%)
Vulva + perineal region	-	-	1	(2.7%)
Uterine cervix + vulva + vagina	1	(8.3%)	3	(8.2%)
				
**Type of HPV Infection**				
Single	6	(50%)	13	(35.2%)
Double	1	(8.3%)	14	(37.8%)
Multiple	5	(41.7%)	10	(27%)
				
**Type of delivery**				
Vaginal	5	(41.7%)	18	(48.6%)
Vaginal + forceps	-	-	1	(2.7%)
Cesarean section	7	(58.3%)	18	(48.6%)
Mean of the gestational age at delivery in the newborn HPV-DNA positive group (39.3 ± 0.9 weeks)				
Mean of the gestational age at delivery in the newborn HPV-DNA negative group (39.3 ± 2.4 weeks)				
				
**Gestational age at the time HPV infection was diagnosed (weeks)**				
≥ 4 to ≤ 12	4	(33.3%)	15	(40.5%)
≥ 13 to ≤ 28	4	(33.3%)	8	(21.6%)
≥ 29 to ≤ 42	2	(16.7%)	6	(16.3%)
Prior to pregnancy	2	(16.7%)	8	(21.6%)
Mean in the newborn HPV-DNA positive group (14.6 ± 13.6 weeks)				
Mean in the newborn HPV-DNA negative group (13.3 ± 12 weeks)				
				
**Time of RUPREME**^3^**(min)**				
≤ 360	12	(100%)	34	(91.9%)
≥ 361 to ≤ 720	-	-	1	(2.7%)
≥ 721	-	-	2	(5.4%)
Mean of newborn HPV-DNA positive group (39 ± 56 minutes)	-	-	-	-
Mean of newborn HPV-DNA negative group (103 ± 240 minutes)	-	-	-	-
				
**Time of labor (min)**				
≤ 240	6	(50%)	22	(61.1%)
≥ 241 to ≤ 360	3	(25%)	9	(25%)
≥ 361	3	(25%)	6	(13.9%)
Mean of newborn HPV-DNA positive group (213 ± 225 minutes)	-	-	-	-
Mean of newborn HPV-DNA negative group (194 ± 196 minutes)	-	-	-	-
				
**HPV lesion at delivery**				
Yes	7	(58.3%)	19	(51.4%)
No	5	(41.7%)	18	(48.6%)

Data are reported as number and percentage (in parentheses) of newborn and children infection positive or negative for human papillomavirus. *P < 0.043 indicates a statistically significant difference between the positive and negative groups by Pearson's chi-square test. ^1^Low-grade squamous intraepithelial lesions. ^2^High-grade squamous intraepithelial lesions. ^3^RUPREME = rupture of amniotic membrane.

### HPV detection and typing methods

Evaluating the HPV-DNA detection and typing methods it was observed that the multiplex PCR methodology identified HPV-DNA in 41 pregnant (83.7%, n = 41/49). In 31 pregnant women (75.6%, n = 31/41) only a single type of HPV-DNA was identified, and in 10 pregnant women (24.4%, n = 10/41) two or more types of HPV-DNA. The nested multiplex PCR method (although used for identification and typing of 9 types of HPV-DNA represented as the most prevalent in the city of Caxias do Sul) identified HPV-DNA in 43 pregnant women (87.8%, n = 43/49). In 28 pregnant women (65.1%, n = 28/43) only one type of HPV-DNA was identified and in 15 pregnant women (34.9%, n = 15/43) two or more types of HPV-DNA. Together the multiplex PCR and nested multiplex PCR methods identified HPV-DNA in 49 pregnant women (100%, n = 49/49). In 19 pregnant women (38.8%, n = 19/49) only one type of HPV-DNA was identified and in 30 pregnant women (61.2%, n = 30/49) two or more types of HPV.

The multiplex PCR method identified HPV-DNA only in two newborns or children (13.3%, n = 2/15), while the nested multiplex PCR method identified it in 13 newborns or children (86.7%, n = 13/15).

## Discussion

HPV infection is considered a sexually transmitted disease common in sexually active young women, with an estimated prevalence between 20% and 46%[4,22,23]. In pregnant women the prevalence of HPV infection fluctuates around 25%[24,25]. Prior studies suggested that the HPV infection could be transmitted during the perinatal period [26-28]. This is a study between mother and newborn, whose type specific HPV agreement between the pair characterized the vertical transmission of the virus.

The presence of HPV-DNA in the maternal genital area may be considered a risk factor for fetal exposure to the virus. In this study, among the types of HPV identified in the maternal genital samples, 54.9% were considered a high carcinogenic risk. HPV infection has been identified in 1% to 20%[27,29] of babies newly born to pregnant women who do not show any evidence of cervical HPV infection, and in 5% to 72%[30,31] in newborns of women with a diagnosis of the viral infection during pregnancy. Gajewska et al. (2006)[25], detected HPV genital (prevalence of HPV types 6/11 – 18%; HPV type 16 – 13%) in 26% (n = 10/39) of the pregnant women and observed a high percentage (70%) of HPV transmission from mother to neonate. Rice et al. (2000)[32] identified HPV-DNA type 16 in samples of the oral cavity of children aged 3 to 11 years, and related the results to possible perinatal and interfamilial transmission. On concluding, these authors suggested that in future vaccination programs and studies of the different transmission routes of HPV should be introduced.

Sedlacek et al. (1989)[33] showed the presence of HPV-DNA in nasopharyngeal aspirates of newborns delivered by vaginal route to mothers with HPV-DNA in uterine cervix cells. Authors have described the presence of HPV-DNA in amniotic liquid[13], in cord blood[14], in fetuses with malformations[7] and in specimens from first trimester spontaneous abortions[34]. The presence of HPV-DNA in newborns no implies the presence of viral infections but may demonstrate the mechanism by which the virus can be transmitted during the perinatal period.

In the study discussed here, on analyzing the samples of buccal and body scrapings, nasopharyngeal aspirate and arterial cord blood obtained in the first minutes of life, it was observed that 11 NB (22.4%, n = 11/49) were positive for HPV-DNA research, 54.9% of which were considered of high carcinogenic risk. Concordance of the type specific HPV was also observed between mother/NB in 16.3% (n = 8/49) of the pairs, characterizing the possibility of transmission of HPV-DNA intrauterine or during the delivery. The different types of HPV identified among the mother/newborn pairs (6.1%, n = 3/49) can be explained by contamination of the sample or of the PCR technique (unlikely, due to the methodologies used to prevent contamination of the PCR methods), or by infection from multiple types of HPV, or by viral subtypes and/or variants.

This study also showed the efficacy of the multiple sample methodology in the newborn, eliminating false negative results for HPV-DNA research. The results obtained corroborate Mazzatenta et al. (1996)[35] who in their study concluded that a simple sample can have a satisfactory result in clinical screening, even if it is not a reliable method to evaluate the risk of vertical transmission of HPV.

The behavior of the presence of HPV-DNA in newborns can be understood by prospective follow up studies and obtaining repeated samples. The present study suggests that the presence of the HPV-DNA in children of mothers HPV-DNA+ in genital sample fluctuates during the first six months of life. Out of the 11 NB who presented HPV-DNA+, only two children continued HPV-DNA+ in samples obtained from the buccal after the first month of life. Three children (6.1%, n = 3/49) became HPV-DNA+ during the first month of life. When children were evaluated in the 6^th ^month of life, all of them became HPV-DNA negative, even if a new case of a HPV-DNA+ appeared for buccal samples. This "regression" of the presence of HPV-DNA in buccal could be explained by the silent neutralization of antibodies that have migrated transplacentally, from the mother to the fetus, and that are functionally active in neonatal circulation, or contamination by maternal infected cells in NB delivered vaginally and that tend to disappear during the first months of life. This diminished number of HPV-DNA+ children could be explained by the results shown by Kawana et al. (2003)[36]. These authors found type 6 anti-HPV antibodies in the maternal blood and in the blood of newborns of infected mothers. The authors suggested that their finding should be better defined and that their study could be considered an important step to understand the prevention of vertical transmission of HPV. Kaye et al. (1996)[37], Cason et al. (1995)[6], Pakarian et al. (1994)[29] Tenti et al. (1999)[38] demonstrated that the HPV-DNA in children examined at three different moments, tends to become negative between birth and the 6th month of life. Puranen et al. (1997)[9] reported the persistence of HPV throughout the first 3 years of life, although the persistence of oral HPV-DNA was not detected in other follow up studies[27,38].

The new cases of children who were HPV-DNA+ in the buccal at the end of the first (n = 3) and sixth (n = 1) month of life, could be explained by the agreement (100%) of the type specific HPV between mother/NB. This agreement of HPV-DNA suggests that these children may have been exposed to HPV-DNA: during the intrauterine period or during the delivery and that the HPV-DNA was only identified after birth[39]; or during the post-birth period, when caring for the child (interfamilial transmission – mother/child)[40].

One (n = 1/63, 1.6%) child had anogenital warts (HPV-DNA type 6/11) in the 12^th ^month of life. The incidence of anogenital warts in children has increased dramatically since 1990 [41-43]. Before 1990, only 136 cases of anogenital warts had been reported in children, yet between 1990 and 1994, at least 326 additional cases were described [42,43]. The increase in incidence of anogenital warts in children is thought to parallel the increase in incidence of anogenital warts in the adult population[43]. Adams (2001)[44] classification scale for evaluation medical findings of suspected sexual abuse lists anogenital warts/condyloma in a child younger than 2 years of age as a nonspecific finding for sexual abuse-perinatal transmission must be considered. Vertical transmission can occur through the bloodstream prior to birth, or at the time of birth, as the infant passes through the infected birth canal. Delivery via cesarean section does not eliminate the possibility of vertical transmission of HPV; there are reports of congenital condyloma after cesarean section without premature rupture of membranes[43]. HPV can be transmitted no sexually from direct contact with caretaker contaminate with genital HPV or common warts [41,43]. For example, caretakers with genital warts who touch or scratch their genitals and then without washing their hands change a baby's diaper or assist a child with toileting/bathing may transmit the virus to the child's genitals. A caretaker with common warts of the hands could transmit HPV in the same manner. HPV transmission via contact with contaminated objects or surfaces is possible [43]. Sexual abuse must never be eliminated when considering possible modes of transmission for anogenital HPV in younger children[42].

In three cases no concordance of the type specific HPV between mother/NB were observed. The different types of HPV-DNA identified among the mother/NB pairs can be explained by infection from multiple types of HPV, or by viral subtypes and/or variants.

The positive and significant correlation between presence HPV-DNA+ in the newborn or child and the maternal variables "history of immunodepression" (HIV, p = 0.007) may be related to the special characteristics of the pregnancy, especially to the changes in the hormonal and immunological balance prevailing during this period, which could favor vertical transmission of the virus. In adults, the risk factors for HPV-DNA transmission have been well established. The chances of perinatal transmission and the differences in the known rates of HPV-DNA transmission, are probably more closely related to the viral load of the infected cells than to the risk factors established for HPV infection[38].

Although the nested multiplex PCR methodology is used to identify only 9 types of HPV represented as the most prevalent in the city of Caxias do Sul, it had an excellent performance to identify maternal HPV-DNA, and also considerably increased the total number of pregnant women with infections caused by multiple viruses. In the samples of newborns, the nested multiplex PCR showed its great sensitivity and specificity to identify HPV. The use of that method was also essential to evaluate the agreement of type specific HPV-DNA between the maternal/newborn samples, thus defining the perinatal transmission rates.

The perinatal transmission of HPV-DNA was suggested when concordance of the type specific HPV was observed between mother/NB and mother/child: eight newborns, three children at the end first month of life and a child in the 6^th ^month of life. In conclusion, perinatal transmission of HPV-DNA was suggested in 24.5% (n = 12/49) of the newborns of mothers with genital warts or intraepithelial lesions of the uterine cervix. Thus, a different management can be adopted for each of the different stages (pre-gestational, gestation, delivery, and the first months post partum), both from the diagnostic and therapeutic perspective. Clinical observation of the mother and the newborn must be maintained, and preventive educational measures established for forms of HPV-DNA transmission, besides effective strategies for specific immunization.

## Competing interests

The authors declare that they have no competing interests.

## Authors' contributions

RLR participated in the design of the study, sampling methods and performed the statistical analysis. RLR made substantial contributions to conception and design, acquisition of data and analysis and interpretation of data. EPS has been involved in drafting the manuscript or revising it critically for important intellectual content.

EPS and JM conceived of the study, and participated in its design and coordination. RLR, EPS, JM, KPL and EZ participated in the sequence alignment and drafted the manuscript. JM, KPL and EZ carried out the molecular studies.

RLR, EPS, JM, KPL and EZ have given final approval of the version to be published.
